# Delineation of chicken immune markers in the era of omics and multicolor flow cytometry

**DOI:** 10.3389/fvets.2024.1385400

**Published:** 2024-05-23

**Authors:** Sonja Härtle, Kate Sutton, Lonneke Vervelde, Tina S. Dalgaard

**Affiliations:** ^1^Department of Veterinary Sciences, LMU Munich, Munich, Germany; ^2^Division of Immunology, The Roslin Institute, Royal (Dick) School of Veterinary Studies, University of Edinburgh, Edinburgh, United Kingdom; ^3^Department of Animal and Veterinary Sciences, Aarhus University, Tjele, Denmark

**Keywords:** chicken, leukocytes, flow cytometry, lineages, phenotyping, single-cell biology

## Abstract

Multiparameter flow cytometry is a routine method in immunological studies incorporated in biomedical, veterinary, agricultural, and wildlife research and routinely used in veterinary clinical laboratories. Its use in the diagnostics of poultry diseases is still limited, but due to the continuous expansion of reagents and cost reductions, this may change in the near future. Although the structure and function of the avian immune system show commonalities with mammals, at the molecular level, there is often low homology across species. The cross-reactivity of mammalian immunological reagents is therefore low, but nevertheless, the list of reagents to study chicken immune cells is increasing. Recent improvement in multicolor antibody panels for chicken cells has resulted in more detailed analysis by flow cytometry and has allowed the discovery of novel leukocyte cell subpopulations. In this article, we present an overview of the reagents and guidance needed to perform multicolor flow cytometry using chicken samples and common pitfalls to avoid.

## 1 Introduction

Flow cytometry is a routine method in immunological studies incorporated in biomedical, veterinary, agricultural, and wildlife research and routinely used in veterinary clinical laboratories, albeit not for the diagnostics of poultry disease. The tremendous expansion in immunological reagents for livestock species, especially pigs and cattle, has in part been due to the availability of cross-reactive antibodies developed in the mouse and human field of immunology, as well as dedicated laboratories developing new antibodies (1–4). Although the structure and function of the avian immune system show commonalities with those of mammals, at the molecular level, there is often low sequence homology across species and low cross-reactivity of mammalian immunological reagents. Using commercially available reagents, recent improvement in multicolour antibody panels for chicken cells has resulted in more detailed analysis by flow cytometry and has allowed the discovery of novel leukocyte cell subpopulations (5–8).

Innovations in cytometry, including traditional flow, spectral flow, and mass cytometry, are driving its use for the isolation and analysis of cells for multi-omics research. Flow cytometry and cell sorting are commonly used tools to phenotype cell populations during, for example, infections and vaccine studies, whereas sorting specific cell subsets can be further analyzed using downstream transcriptomics, including bulk RNASeq (9, 10), single cell (sc) (11–13), or single nucleus (sn) sequencing. High-resolution transcriptomics are instrumental to understand avian immunology and contributing to defining accurate biomarker signatures of diseases. Although not yet applied to avian immune cell flow cytometry and cell sorting, they can also be used for single-cell proteomics analysis (14, 15) and have been applied to analyse chicken sensory epithelium (16). The development of flow cytometry combined with omics technologies for avian research is rapidly enhancing and reviewed in Liu et al. (17). Validating scSeq data through flow cytometric analysis or immunohistology strengthens and verifies the data set, and thus a critical review of the single cell analysis should always be part of the quality control of sequence analysis (18). For example, recent studies (18, 19) demonstrated that due to little *de novo* mRNA production, especially avian CD4, is more difficult to detect in scSeq data, and results could be easily misinterpreted if not compared with flow cytometric CD4 staining.

**Figure 1:**
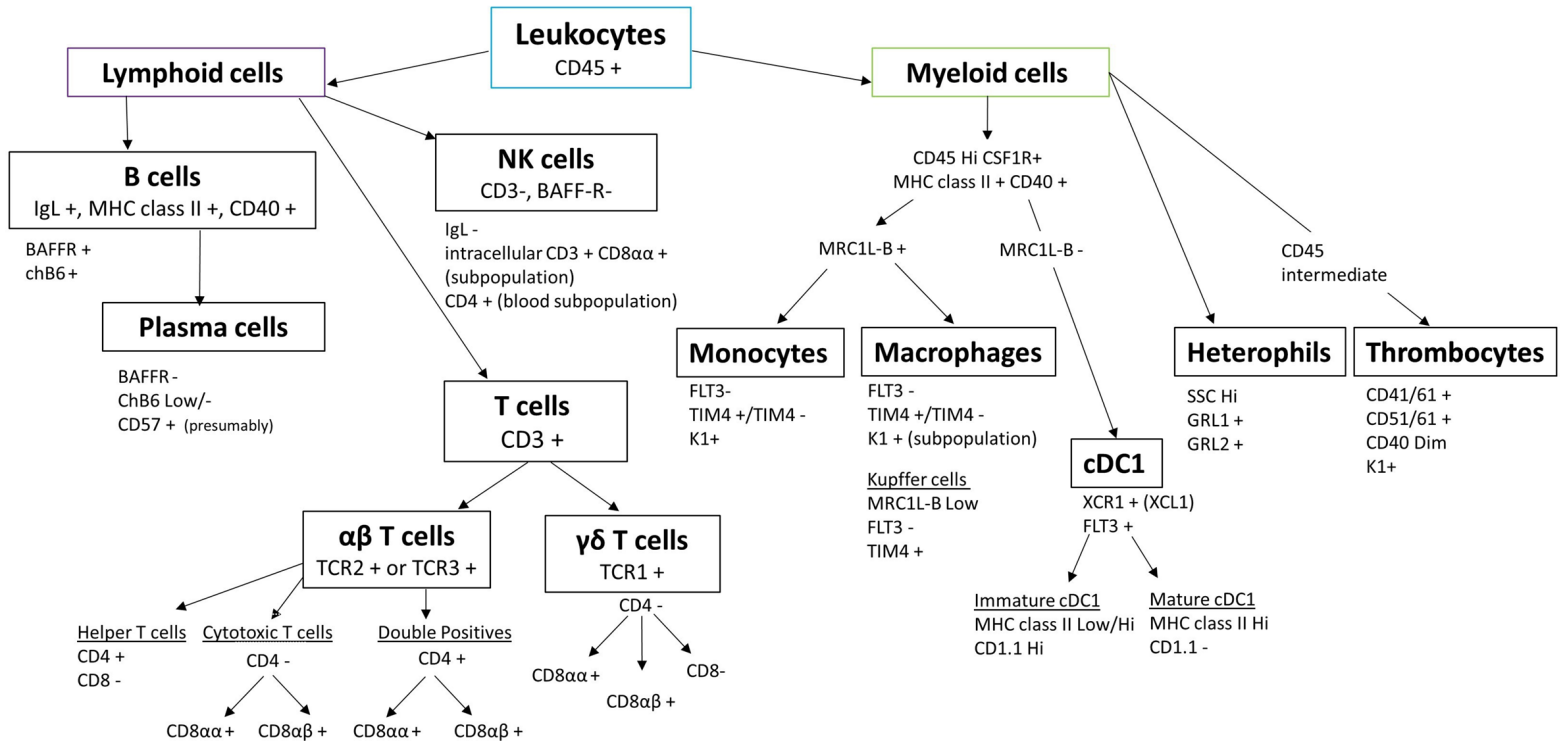
Chicken leukocyte cell lineages and their characteristic markers routinely used in flow cytometry.

The key to robust single-cell preparation is the quality of the cell sample. Sample quality is dependent on multiple steps, including the freshness of the tissue, the digestion step, either mechanical or enzymatic, and the time the preparation takes. These all affect cell viability, the amount of cell debris and aggregates, and the loss of certain cell subpopulations. The advantage of mechanical dissociation is that cell surface antigens are least affected compared to enzymatic dissociation; however, the breakdown of extracellular matrix is difficult for some tissues, such as the lung, intestine, liver, and brain, and isolation of rare cells is less likely. Different digestive enzymes, alone or in combination, can be used to break down extracellular matrix or cell–cell junction, but one method is rarely suitable for different tissues due to the large variation in cellular composition and extracellular matrix composition (17, 20). Whatever the choice of cell preparation, speed is of the essence, and awareness that changes are likely to occur should be taken into account when analysing the data. In addition, cell plasticity is widely accepted, but little is known with regard to chicken immune cells. Cells can change from one phenotype to another, for example, because the cell preparation or purification activates the cells, but also clear-cut delineation of cell subpopulations has proved challenging in livestock species (10, 21). Transgenic chickens represent a great potential to study immune cells in more detail, especially those for which few antibodies or known markers are available, increasing our capacity to distinguish different cell lineages (12, 22). In this article, we present an overview of the reagents and guidance needed to perform multicolour flow cytometry using chicken cells and common pitfalls to avoid. An overview of chicken leukocyte subsets and their delineation by flow cytometric markers is shown in Figure 1.

## 2 CD45—the pan leukocyte marker

**Figure 2:**
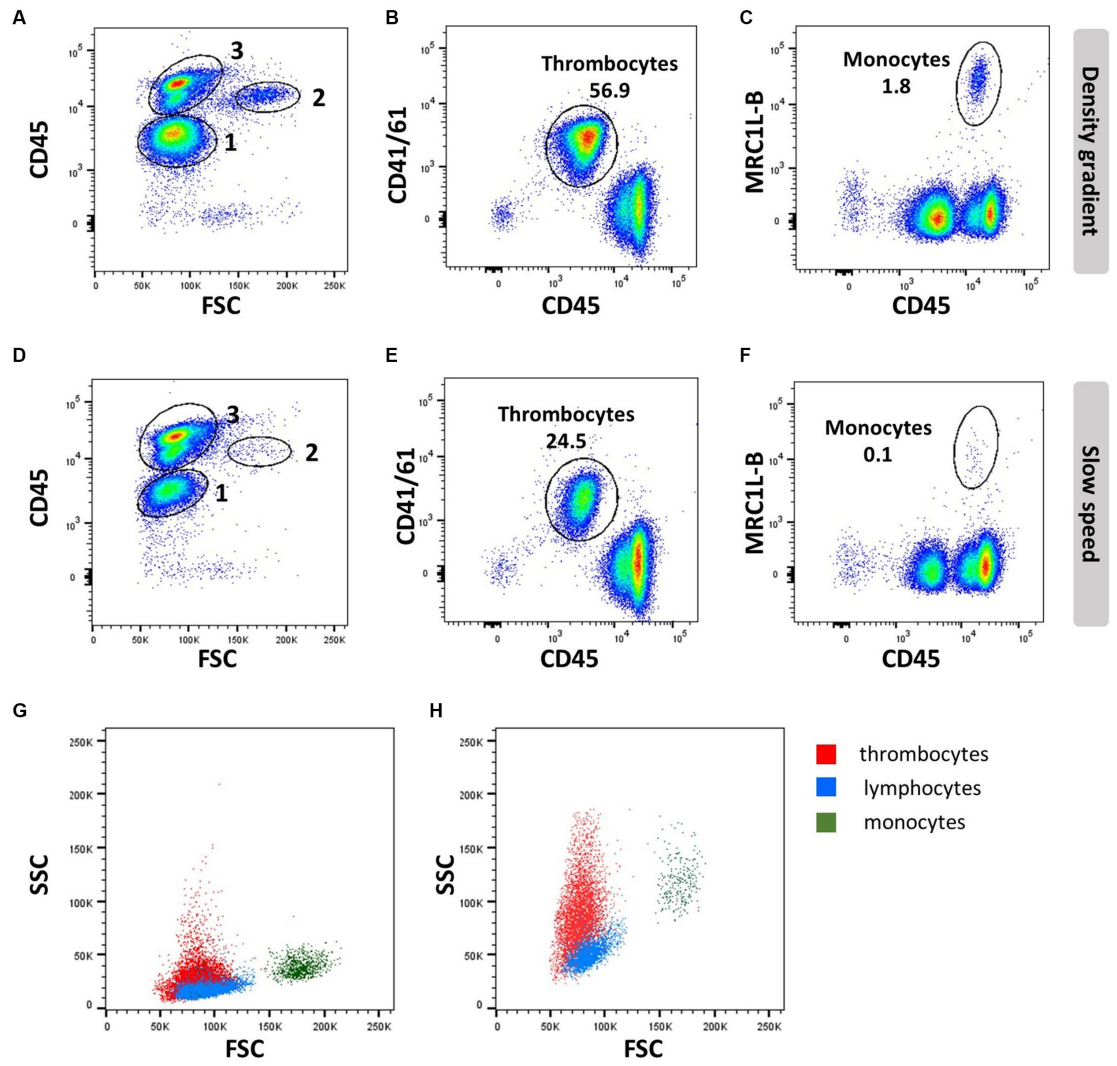
Thrombocyte characteristics: EDTA blood was processed by density gradient centrifugation **(A–C,G)** or slow speed centrifugation **(D–F,H)** and stained with anti-CD45 (16-6), followed by anti-mouse-IgG2a-FITC and anti-CD41/61 (11C3) and anti-IgG1-PE **(A,B,D,E,G,H)** or anti-MRC1L-B (clone KUL01, IgG1) and anti-IgG1-APC **(C,F–H)**. Numbers in scatter plots **(A,D)** represent 1 = thrombocytes, 2 = monocytes, and 3 = lymphocytes; FSC/SSC profile of thrombocytes (red), lymphocytes (blue), and monocytes (dark green) analyzed with common **(G)** or increased SSC voltage **(H)**.

The transmembrane glycoprotein CD45 is a tyrosin phosphatase that regulates a large variety of cellular functions. In mammals, it is expressed in all nucleated cells of haematopoietic origin (23). In chickens, CD45 is expressed on all leukocytes, including thrombocytes, but absent on nucleated cells of the erythroid lineage (24). The expression of CD45 on thrombocytes is significantly lower compared to other leukocytes. Depending on the cell isolation procedure, antibody, and staining protocol used, additional distinction between B cells and myeloid cells (medium expression) and T cells (high expression) can also be observed (see Figure 2) (7). Several anti-chicken CD45 mAbs are commercially available, such as LT40 (IgM), AV53 (IgG1), UM16-6 (IgG2a), and His C7 (IgG2a) (Table 1). Alternative splicing of mammalian CD45 leads to the expression of isoforms of different lengths, which are named according to the presence or absence of exons 4 (A), 5 (B), and 6 (C) (CD45RO, CD45RA, and CD45RB, respectively) (25). Expression of the isoforms varies between cell types and subsets and depends on the cellular differentiation and activation state (26, 27). For chicken CD45, expression of different isoforms caused by alternative splicing of exons 3, 5, and 7 was also demonstrated (28, 29). Whilst the above-mentioned chicken CD45 mAbs detect all isoforms, mAb 8B1 (IgM) recognises only two different short isoforms, which exhibit different expression patterns on B cells, αβ T cells, and γδ T cells (29). As activation of γδ T cells upregulated the expression of CD45 short isoforms, a varying expression system similar to that of mammals could exist in chickens, and the mAb 8B1 would be a helpful tool for its analysis. Antibody clones recognising CD45 and other relevant surface markers for leukocyte delineation are listed in Table 1.

**Table 1:** Antibodies against surface markers, which are mentioned in the text.

**Antigen**	**Clone**	**Isotype**	**Availability**	**Comments**
Cath2	N/A	Rabbit polyclonal		
CD1.1	CB3	IgG1	Southern Biotech	
CD107a	5G10	IgG1	DSHB	
CD115 (CSF1R)	ROS-AV170	IgG1	Bio-Rad	
CD127 (IL7R)	8F10E11	IgM		Steric hindrance with CD4 when co-staining
CD15	W6D3	IgG1	BioLegend	
CD15s	CSLEX1	IgM	BD Biosciences	
CD184 (CXCR4)	9D9	IgG2a	Bio-Rad	
CD185 (CXCR5)	6A9	IgG1	S. Härtle LMU	
CD25 (IL2R-alpha)	20E5	IgG1	T. Göbel LMU	
	28–4	IgG3	T. Göbel LMU	
	AbD13504	HuCAL Fab	Southern Biotech	
	AV142	IgG1	Bio-Rad	
CD268 (BAFF-R)	2C4	IgG1	Bio-Rad	
CD28	AV7	IgG1	Southern Biotech	
CD3	CD3-12	IgG1	Thermo Fisher	
	CT-3	IgG1	Bio-Rad/Southern Biotech	
	AV36	IgG1	Immunological Toolbox	Does not bind to T cells from NARF C.B12 line
CD4	CT4	IgG1	Southern Biotech	
	EP96	IgM	Southern Biotech	
	2.35	IgG2b	Bio-Rad	
	AV29	IgG2b	RI Immunological Toolbox	
	AV30	IgG1	RI Immunological Toolbox	
CD40	IG8	IgG2a	Immunological Toolbox	
CD41/CD61	11C3	IgG1	Bio-Rad	
CD44	AV6	IgG1	Bio-Rad/Southern Biotech	
CD45	LT40	IgM	Southern Biotech	Lower discrimination between thrombocytes and lymphocytes compared to other clones
	UM16-6	IgG2a	v	
	AV53	IgG1	RI Immunological Toolbox	
	His C7	IgG2a	WUR NL	
CD45 (2 short isoforms)	8B1	IgM	T. Göbel LMU	
CD5	2–191	IgG1	Discontinued	
CD51/CD61	23C6	IgG1	Thermo Fisher/BD Biosciences/BioLegend	
CD56	4B5	IgG1	T. Göbel LMU	
CD57	HNK-1	IgM	BioLegend	Crossreacting human antibody
CD80	AV82	IgG2a	Immunological Toolbox	
CD8α	11.39	IgG1	Bio-Rad	
	3–298	IgG2b	Southern Biotech	Superior clone recognising the majority of CD8α variants
	CT-8	IgG1	Southern Biotech	
	AV12	IgG1	Immunological Toolbox	
	AV13	IgG1	Immunological Toolbox	
	AV14	IgG2b	Immunological Toolbox	
	EP72	IgG2b	Southern Biotech	
CD8β	EP42	IgG2a	Southern Biotech	
chB6/Bu-1	AV20	IgG1	Bio-Rad	B cells but also some IELs, subpopulation of MCR1L-B macrophages
chB6a/Bu-1a	21-1A4	IgG1	Thermo Fisher	
	L22	IgG1	Bio-Rad	
chB6b/Bu-1b	5-11G2	IgG1	Thermo Fisher	
	15H6	IgG1	Southern Biotech	
ChL12/OV	11A9	IgM	S. Härtle LMU	
CLEC-2	8G8	IgG2a	T. Göbel LMU	
FcY/CHIR-AB1	8D12	IgG2b	T. Göbel LMU	
FLT3	ROS-AV184	IgG1	RI Immunological Toolbox	High on dendritic cells and low on subpopulation of MRC1LB+ macrophages
GRL1	I-A5	IgG3	DSHB	
GRL2		IgG1	DSHB	
Ig Light chain	2G1	IgG1	Bio-Rad	
	L1	IgG1	VWR/GeneTex	
IgA	A1	IgG2b	Southern Biotech	
IgM	M1	IgG2b	Southern Biotech	
IgY	4D12	IgG1	Bio-Rad	Optimal use for ELISA/immunohistology not suitable for flow
	G1	IgG1	Southern Biotech	Detects membrane and soluble IgY
MHC I	F21-2	IgG1	Southern Biotech	
MHC I (beta 2 microglobulin)	F21-21	IgG1	Southern Biotech	
MHC II	2G11	IgG1	Southern Biotech	
	TAP1	IgG2a	DSHB	
MRC1L-B	KUL01	IgG1	Southern Biotech	
Putative CD11c	8F2	IgG1	S. Härtle LMU	
SLAMF4	8C7	IgG1	T. Göbel LMU	
TCR αβ1 (Vβ1)	TCR2	IgG1	Southern Biotech	
TCR αβ2 (Vβ2)	TCR3	IgG1	Southern Biotech	
TCRγδ	TCR1	IgG1	Southern Biotech	
TIM4	JH9	IgG1	RI Immunological TOOLBOX	
TREM-A1	14C9	IgM	T. Göbel LMU	
TREM-B1	7E8	IgG1	T. Göbel LMU	
	1E9	IgG2a	T. Göbel LMU	
Unknown	K1	IgG2a	B. Kaspers/S. Härtle LMU	

## 3 Thrombocytes

Thrombocytes are the most common white blood cell population in the circulation of chickens, making up to ~80% of circulating peripheral blood mononuclear cells. In contrast to mammalian cells, avian thrombocytes are nucleated cells that display a variety of immunological functions, such as phagocytosis and tissue repair, and can release an array of bioactive proteins, including cytokines. The type of anticoagulants and isolation procedures affect the viability and number of thrombocytes; they can be isolated by PBL gradient centrifugation (Figures 2A–C), whilst slow spin or differential centrifugation (60–100× *g*) results in a major loss of thrombocytes (Figures 2D–F) (30, 31).

Differentiation of thrombocytes from lymphocytes based on morphology is difficult, although thrombocytes are slightly smaller with clear cytoplasm and more oval to spindle-shaped. Compared to erythrocytes, they are smaller and have a more rounded nucleus and an increased nucleus-to-cytoplasm ratio (32). These cellular properties present as low forward side scatter (FSC) similar to lymphocytes but a higher side scatter (SSC) than lymphocytes, whereas monocytes have a higher FSC and SSC compared to lymphocytes (Figures 2A,D,G,H).

Avian thrombocyte surface markers have been identified and facilitate experimentation using flow cytometry mAbs specific for alpha IIb beta 3 integrin (GpIIb/IIIa complex or CD41/CD61; clone 11C3), and CD45 will distinguish thrombocytes from leukocytes based on their CD45^Low^ CD41/CD61^+^ phenotype (Figures 2B,E). However, CD41/61 is not exclusive for thrombocytes (33). Chicken TREM-B1 (mAb clones 7E8 and 1E9), an inhibitory receptor, is exclusively expressed in thrombocytes. The C-type lectin receptor CLEC-2 (mAb clone 8G8) (34) can be used in combination with CD8α and K1. The molecule that is recognized by mAb K1 has not been identified, but it is expressed in thrombocytes, macrophages, and monocytes. The thrombocytes can be distinguished based on size, smaller than the macrophages and monocytes, if a single cell gate is applied. In addition, thrombocytes express a CD51/CD61 integrin on their surface as well as a signaling lymphocyte activation molecule (SLAM)F4 (35), TREM-A1 (33), CD40 (36) and MHC I (37). However, these markers have to be used in a multicolour panel to distinguish the thrombocytes from leukocytes, and in addition, a marker such as SLAMF4 is only expressed in a subpopulation of thrombocytes (35). Although it has been reported that thrombocytes express MHC II mRNA (38), accurate demonstration of MHC II surface expression is lacking so far.

## 4 Polymorphonuclear cells

Chickens have limited numbers of eosinophils and mast cells, and the dominant polymorphonuclear cell type is the heterophil. To the best of our knowledge, no specific flow cytometry-applicable surface markers for granulocyte subsets are available in chickens. Heterophils have been reported to lack myeloperoxidase activity; however, older literature (39) and immunocytochemical staining (40) suggest there is peroxidase activity that in the future may be detected by cross-reactive antibodies. The mAbs, anti-GRL1 and anti-GRL2, stain the granules of chicken granulocytes and thrombocytes, in addition to the surface expression of these proteins due to exocytosis (41). Increased expression can therefore be detected after permeabilisation of the cells, staining both surface and intracellular GRL1 and GRL2. Surface expression of GRL2 can also be found on activated T cells (18). Heterophils also express antimicrobial peptides, including the cathelicidin CATH-2, which can be stained with a rabbit polyclonal serum (40), which in principle could be used for intracellular staining and flow cytometry. The most applicable method to detect heterophils is through a high SSC pattern and the lack of expression of B-cell, T-cell, thrombocyte, and macrophage markers in the CD45^+^ population. The high SSC is a consistent feature of heterophils, but the FSC pattern has been shown to be dependent on the flow cytometry equipment and the software used to analyse the data (7, 42).

Eosinophils are found in peripheral blood based on the staining of eosinophilic granules in their cytoplasm. However, chickens lack IgE isotypes and components of allergic reactions, which make it questionable if these eosinophils are functionally comparable to mammalian eosinophils. Whilst heterophils have a high SSC and low FSC pattern, eosinophils have a low SSC and higher FSC pattern (7), but a lack of specific markers hampers quantitative analysis by flow cytometry. Eosinophils have been reported to also have endogenous peroxidase activity (43), but the use of peroxidase activity in flow cytometry for chicken cells has not been demonstrated. Like eosinophils, detecting mast cells by flow cytometry is problematic due to a lack of markers. Cells containing Alcian Blue-positive granules in the lamina propria of the intestinal tract have been described (44) but the flow cytometric analysis of granulocyte subsets remains limited.

## 5 Natural killer cells

**Figure 3:**
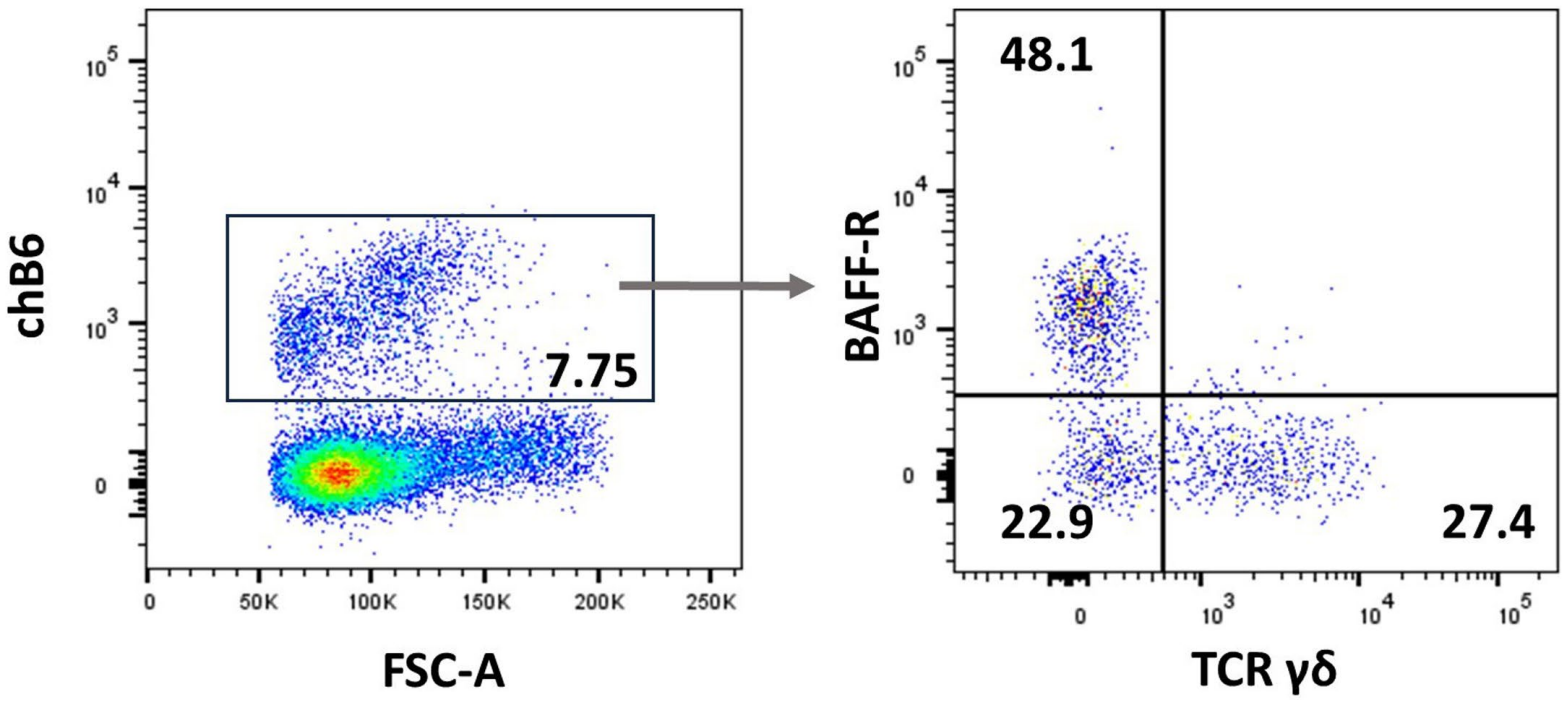
chB6 expression on non-B cells: an IEL preparation was stained with antibodies against chB6 (AV20), TCRγδ (TCR1), and BAFF-R (2C4). Cells were gated for viable, single leukocytes. Gating on chB6^+^ cells **(A)** reveals that chB6^+^ cells consist of three subsets: BAFF-R^+^/TCRγδ^−^ B cells, BAFF-R^−^/TCRγδ^+^ γδ T cells, and so far, uncharacterised BAFF-R^−^/TCRγδ^−^ cells **(B)**.

Natural killer (NK) cells display many different inhibitory and activating receptors that mediate a variety of functions, from the classical role of killing pathogen-infected cells to regulatory functions influencing adaptive immunity through interactions with dendritic cells (DCs) and secretion of cytokines. Chicken NK cells have been described in the embryonic spleen before the T cells enter the periphery. These cells are CD45^+^ and lack T-cell or B-cell-specific markers on the cell surface, i.e., surface CD3^−^, BAFF-R^−^, and Ig Light chain^−^, but CD3 is detected intracellularly (45). The number of NK cells in peripheral blood is low, whereas more substantial numbers can be found in tissues. Two inconsistencies in the avian NK cell literature should be highlighted before describing the recent flow cytometric data. Firstly, the mAb clone 28–4 has been used to detect chicken NK cells in the intestinal epithelium for many years, until more recently, it was shown to detect IL2Rα (CD25) (46). Therefore, although very useful in a multicolour panel, the antibody is not specific for NK cells. Secondly, chB6 has been used as a B-cell marker for decades (47). However, it is also expressed on intraepithelial leukocytes in the intestine that are CD45^+^ CD3^−^ Ig Light chain^−^ and lack markers expressed on mononuclear phagocytes (Figure 3) (44).

Many antibodies have been tested to identify chicken NK cells, but none were shown to be uniquely expressed by NK cells, as these also detect subpopulations of T cells, thrombocytes, or myeloid cells (48–51). Alternatively, they only detect a subpopulation of NK cells [reviewed in Straub et al. (52)]. Expression of receptors also varies between tissues and subpopulations of NK cells in the lung, liver, and intestinal epithelium (52). These include antibodies specific for CD56 (49) and CHIR-AB1 (53), a high-affinity IgY Fc receptor. Nonetheless, they are useful for flow cytometry in multicolour panels in combination with the lack of surface CD3, BAFF-R, or Ig Light chain staining (48). Chicken NK cells that are CD3^−^ Ig Light chain^−^ can express CD8αα, but the expression level alters upon activation, and expression on NK cells in peripheral blood may vary. A NK cell-like population in peripheral blood was detected, which expressed low levels of CD4, CD5, and CD11c and high levels of CHIR-AB1, CD56, and 28–4 but lacked CD3, CD8α, and chB6 (54).

To measure NK cell function, a flow cytometry-based degranulation assay can be applied that is based on the expression of CD107a (LAMP-1 or LEP100) (48). Cytotoxic activity via the perforin-granzyme pathway occurs in pre-formed lytic granules surrounded by lipid bilayers containing LAMPs that are fused with the plasma membrane. A chicken homolog of LAMP-1 (CD107a) exists (also known as LEP100), and a mAb antibody (5G10) is available, which was first used to assess the degranulation of chicken NK cells (48) and then applied to study CTLs (55). To distinguish between NK cells and CTLs, this staining must be combined with additional mAbs to exclude degranulation of cytotoxic T cells (55) and heterophils (48). More recently, flow cytometric-based staining of the release of perforin and granzyme A was developed to measure NK cell activation in ED14 embryonic splenocytes (56), but like CD107a expression, both perforin and granzyme A are not restricted to NK cells, and multicolour analysis is warranted to exclude CTLs. The lack of exclusivity is also demonstrated by perforin and granzyme A expression in the macrophage cell line HD-11 and low levels of perforin expression in the B-cell line DT-40 (56), which is in agreement with expression in human macrophages and granzyme B secretion by human B cells.

## 6 Mononuclear phagocytic cells

Similar to mammals, the cells of the chicken mononuclear phagocytic system (MPS) consist of monocytes, macrophages, and dendritic cells (DC). Studies into the biology of these cells using flow cytometry have focused on cells of the blood, spleen, and occasionally the liver and lung. In the blood, monocytes can be detected using the antibody KUL01, which recognises the mannose receptor C-type 1 Like B (MRC1L-B (57)). In whole blood cell preparation, monocytes are characterized by their high FSC and low SSC compared to lymphoid cells (7). MRC1L-B^+^ monocytes also express CSF1R and MHC class II (22, 58).

TIM4 binds phosphatidylserine, a lipid normally found on the inner surface of the plasma membrane that is rapidly redistributed to the outer cell surface during apoptosis (59). Like humans, chickens express short and long isoforms of TIM4. Hu et al. generated two monoclonal antibodies against chicken TIM4. Clone JH9 was raised against the extracellular domain of TIM4 and recognises all TIM4 isoforms, whilst clone IE12 was raised against the additional linker found in some of the TIM4 long isoforms. This clone only recognises one of the long TIM4 gene products (available through the Roslin Institute, Immunological Toolbox). Currently, there is no known functional difference between the chicken TIM4 isoforms, but differential expression at the mRNA level appears to be specific to chicken lines (60). Staining chicken leukocytes with the TIM4 mAb JH9 does not provide a distinct staining pattern in flow cytometry, and it is therefore challenging to distinguish clear boundaries between TIM4^+^ and TIM4^−^ populations without correct unstained and FMO controls. However, in combination with MRC1L-B, the antibody has been useful in the identification of subpopulations of cells in chickens.

In chickens, the presence of monocyte subsets described in mammals, such as the classical CD14^++^ CD16^−^ (mouse equivalent LyC6^++^ CD43^+^), the non-classical CD14^++^ CD16^++^ (LyC6^+^ CD43^++^), and intermediate CD14^++^ CD16^+^ (LyC6^++^ CD43^++^) monocytes (61), cannot be clearly defined due to a lack of antibodies against these markers. Unlike mammalian CD14, chicken CD14 is a GPI-anchored protein rather than a transmembrane protein (62). To date, no specific staining has been demonstrated for the mAb anti-chicken CD14. However, chicken monocytes can be segregated based on their expression of TIM4. MRCL1-B^+^ TIM4^+^ and MRCL1-B^+^ TIM4^−^-cell populations both express transcripts for genes involved in murine monocyte–macrophage differentiation, indicating these cells are part of a differentiation series rather than distinct subsets (63). For *in vitro* characterization of monocytes, cells can be enriched by their adherence to glass or plastic tissue culture plates. Studies have shown that monocytes can adhere to glass after 1 h of culture. However, nucleated thrombocytes attach to these surfaces within 30 min but these cells die within 48–72 h; therefore, monocytes cultured for shorter periods of time will be contaminated with thrombocytes (64).

### 6.1 Macrophages

Chicken macrophages can be universally studied in tissues by flow cytometry using the MRC1L-B antibody (9, 10, 60, 65, 66). Tissue-resident macrophages exhibit diverse functionality and can be defined based on their location in the organ. For example, in the chicken spleen, several macrophage subpopulations exist. These include periarteriolar lymphoid sheaths, resident macrophages, ellipsoid-associated macrophages, and red-pulp macrophages. The ability to segregate different macrophage subsets is difficult as specific markers for each population have yet to be identified. Splenic MRC1L-B^+^ macrophages universally express MHC class II, CD40, and CD80 and lack FLT3 expression (10, 67). Although mAbs against chicken CD83 and CD86 have been described, no convincing staining of mononuclear phagocytes has been demonstrated. In the liver, MRC1L-B^+^ macrophages can be segregated into MRC1L-B^Low^ TIM4^Hi^ cells and MRC1L-B^Hi^ TIM4^Low^ and MRC1L-B^Hi^ TIM4^−^ cells. Transcriptome analysis indicates that MRC1L-B^Low^ TIM4^Hi^ represent Kupffer cells, which are highly phagocytic compared to the MRC1L-B^Hi^ liver-resident macrophages (63). The TIM4 mAb JH9 stains a small population of CD3^+^ and Bu1^+^ cells in the liver and bursa, respectively (63).

Functional assays involving the assessment of phagocytosis can be integrated into flow cytometry experiments. Using commercially available fluorescent beads, which can be labeled with antigens such as LPS or inactive avian influenza virus, pH-sensitive pHrodo-labeled bioparticles, such as *Salmonella* or *E. coli*, or CFSE-labeled dead cells, can be utilized to determine the efficiency and specificity of chicken macrophage phagocytosis or effectorcytosis (10, 68–70). Performing phagocytosis assays at 4°C, a temperature commonly referred to as “on ice,” should be used to assess specific binding or adhesion of particles to cell surfaces without allowing active internalization (phagocytosis) to occur. This approach helps researchers differentiate between particles that are merely attached to the cell membrane and those that have been engulfed by the cell. It should be noted that 4°C control may not always be optimal for *in vitro* model antigen uptake studies. For example, 4°C control does not prevent bone marrow-derived macrophages from phagocytosing pH-sensitive pHrodo-labeled bioparticles. Instead, an actin polymerization inhibitor, cytochalasin D, inhibited the uptake of these bioparticles (70). The differential expression of surface markers, such as CD40 and MHC class II, can be an indicator of cell activation. In chickens, LPS-treated bone marrow-derived macrophages upregulate CD40 expression and downregulate MHC class II expression (71). In the chicken lung, MRC1L-B^+^ that phagocytosed LPS- or avian influenza-coated beads had significantly higher CD40 expression compared to cells that had taken up uncoated beads. The same study also demonstrated an increase in MHC class II expression by cells that phagocytosed LPS-coated beads (72). This observation is still to be determined for other tissue-resident macrophages, and more research is required to understand how infection alters the expression of these markers in a tissue-specific manner.

**Figure 4:**
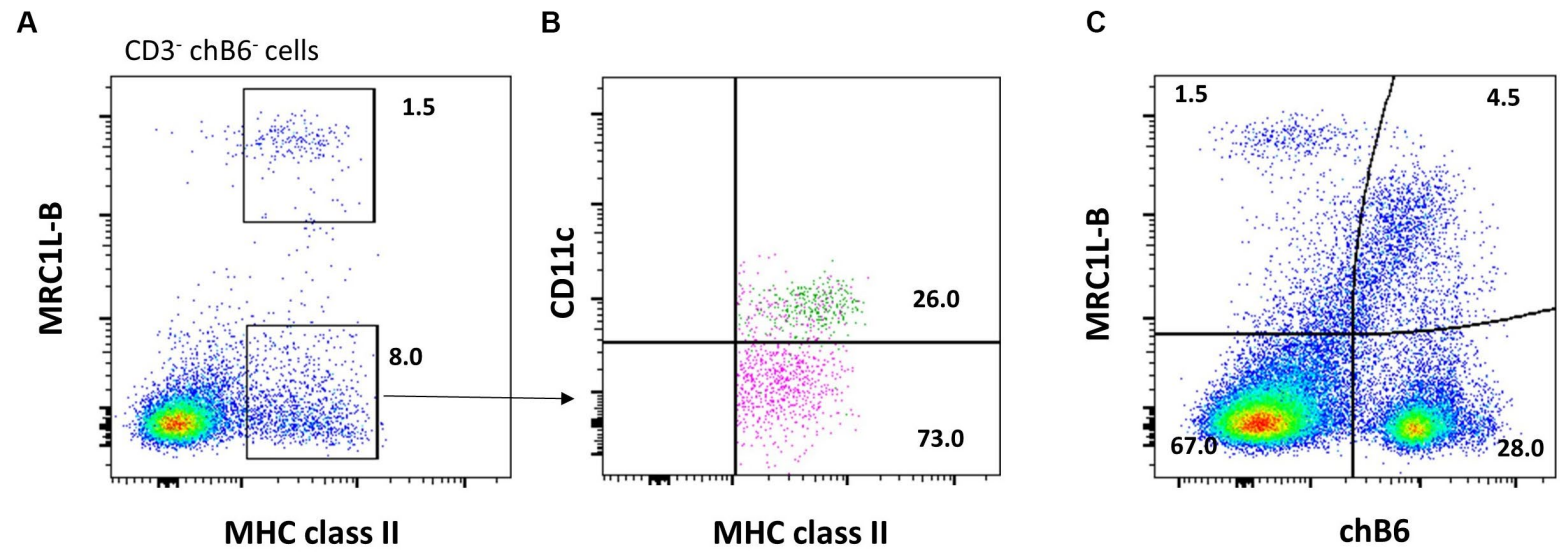
Enriching for splenic cDC1 cells without the FLT3 antibody. Splenocytes were stained for CD3 (CT3), chB6 (AV20), MRC1L-B (KUL01), MHC class II (Tap1), and putative CD11c (8F2). **(A)** Cells gated for live, single CD3^−^/chB6^−^ leukocytes, the CD3^−^ chB6^−^ cell population can be analyzed according to MHC class II and MRC1L-B expression. **(B)** Amongst the CD3^−^ chB6^−^ MRC1L-B^−^ MHC class II^+^ cells, CD11c ^+^ cDCs (green) can be addressed. **(C)** ChB6 (AV20) antibody stains MRC1L-B^+^ macrophages in the spleen.

It has been well known that a small population of chicken splenic macrophages stain for chicken B-cell marker chB6 (Figure 4C) (47). Therefore, the BAFF-R mAb is a more specific reagent for chicken B cells (73). Of note, MRC1L-B may be sensitive to enzymatic digestion. To assess the impact of isolation techniques on MRC1L-B expression, researchers should examine immunohistology sections of their tissue samples to visualize the level/abundance of cells expressing the marker. Specifically, comparing the effects of non-enzymatic approaches to enzymatic methods can provide insights into how different isolation techniques influence MRC1L-B expression.

### 6.2 Dendritic cells

Generally, in mammals, DCs are defined by their expression levels of MHC class II, CD11c, and co-stimulatory molecules CD40 and CD86 (74). Using this general phenotype, researchers have sought to phenotype chicken DCs in this manner by flow cytometry. Vu Mahn et al. found that MHC class II^+^ putative CD11c^+^ MRC1L-B^−^ splenocytes express gene transcripts associated with mammalian cDC1 cells (75). The antibody against DEC-205, a marker for murine DC, was generated for chickens. Although useful in immunohistology, this antibody does not provide a strong staining pattern in flow cytometry (76). Recently, reagents against chicken FLT3, XCR1, and CSFR2 were developed, which aid to study chicken cDC without dependence on transgenic chickens (67). The anti-chicken FLT3 monoclonal, designated ROS-AV184, was found to label two cell populations in the spleen, FLT3^Hi^ and FLT3^Low^ cells. The FLT3^Hi^ cells, known as cDC1 cells, lack expression of the macrophage marker, MRC1L-B, and exhibit slightly lower levels of CD45 and MHC class II expression compared to the FLT3^Low^ cells. The FLT3^Low^ cells express MRC1L-B, making them macrophages. If receptor–ligand interaction is of high affinity, this offers an opportunity to analyse protein expression on cells through flow cytometry with fluorochrome-labeled ligands instead of antibodies. Recently, Wu et al. demonstrated that chicken XCL1-AF647 binds to XCR1 on FLT3^+^ cDC1 cells. In addition, to detect CSF2R expression, a CSF2-AF647 protein was generated and found not to stain chicken cDC1 cells (67). Together, this demonstrates that chicken cDC1 cells can be distinguished by staining for FLT3 or XCR1. However, if researchers do not have mAb FLT3, DC can be enriched by including MRC1L-B, CD3, and chB6 or BAFF-R staining with MCH class II to remove T and B cells and macrophages from MHC class II^+^ cell population (Figures 4A,B).

The maturation status of chicken cDC1 can be defined by their expression of CD1.1 using mAb CB3 clone (77). In the blood, a majority of XCR1^+^ cDC1 are MHC class II^Low^ and CD1.1^Hi^, whereas small subpopulations have the MHC class II^Hi^ CD1.1^Hi^ or MHC class II^Hi^ CD1.1^−^ phenotype. In the spleen, these subpopulations are present from 1 week of hatch, with the MHC class II^Hi^ and CD1.1^−^ cells becoming the most abundant by 2-week post-hatch. It is hypothesized that the splenic XCR1^+^ MHC class II^Hi^ CD1.1^Hi^ cDC1 is derived from the blood XCR1^+^ MHC class II^Hi^ CD1.1^Hi^ cDC1 pool that lose CD1.1 expression as they mature and develop in the spleen (12).

## 7 B cells

Chickens use the bursa Fabricii, a gut-associated lymphoid tissue (GALT), to expand B-cell precursors and diversify the BCR repertoire. This unique primary B-cell organ is the most striking difference to B-cell development in most mammals and causes the classification of avian B-cell development into a pre-bursal, bursal, and post-bursal phase, resulting in the discrimination of pre-bursal, bursal, and post-bursal B cells (78).

### 7.1 Pre-bursal B cells

The earliest B-cell-specific surface marker expressed on pre-bursal B cells is Bu1/chB6, with the first chB6^+^ cells becoming detectable simultaneously around embryonic day (ED) 10 in the embryonic bursa and spleen (79). As chB6 is strongly expressed at all stages of B-cell development except in differentiated plasma cells (47), it has become the most used marker for chicken B cells. The protein is a typical type I transmembrane protein with a highly glycosylated extracellular region and no recognizable similarity to known mammalian molecules. chB6 is recognized by several commercially available mAbs like AV20 and BoA1 (a cross-reactive guinea fowl antibody). It is an alloantigen with two alleles, Bu1a/chB6a and Bu1b/chB6b, which are recognized by anti-chB6a (clone L22) and anti-chB6b (clone 11G2) (80, 81). If using the allotype-specific antibodies to address all B cells, it is important to determine the presence of the alleles in the chicken line; otherwise, only a fraction of B cells might be stained.

Shortly after chB6 expression becomes detectable, pre-bursal B cells begin to express another pan-B-cell marker, the BAFF receptor (BAFF-R), recognized by mAb anti-BAFF-R clone 2C4 (73) and at ED14, chB6+ cells in the spleen are all BAFF-R+ (82).

Whilst most cells before their migration to the bursa are Ig-negative, very few pre-bursal cells have completed a productive BCR rearrangement and express surface Ig, detectable with anti-Ig Light chain or anti-IgM staining (82, 83). All pre-bursal B cells in the spleen express relatively high levels of CXCR4 and CXCR5 (82, 84); hence, migration of pre-bursal B cells can be mediated by their attraction toward CXCL12 and CXCL13L1-L3, respectively. Potentially also connected to their migratory behavior, pre-bursal B cells express sialyl-Lewis-X/CD15s but not Lewis-X/CD15 (85, 86). CD15s is a tetrasaccharide carbohydrate that is usually attached to O-glycans on the surface of cells and can mediate the interaction with selectins as the first step of leukocyte emigration from blood vessels. The chicken molecules can be detected with cross-reacting mAbs for the human molecules, e.g., mouse anti-human CD15s clone CSLEX1 and mouse anti-human CD15 clone W6D3 (85, 86).

### 7.2 Bursal B cells

**Figure 5:**
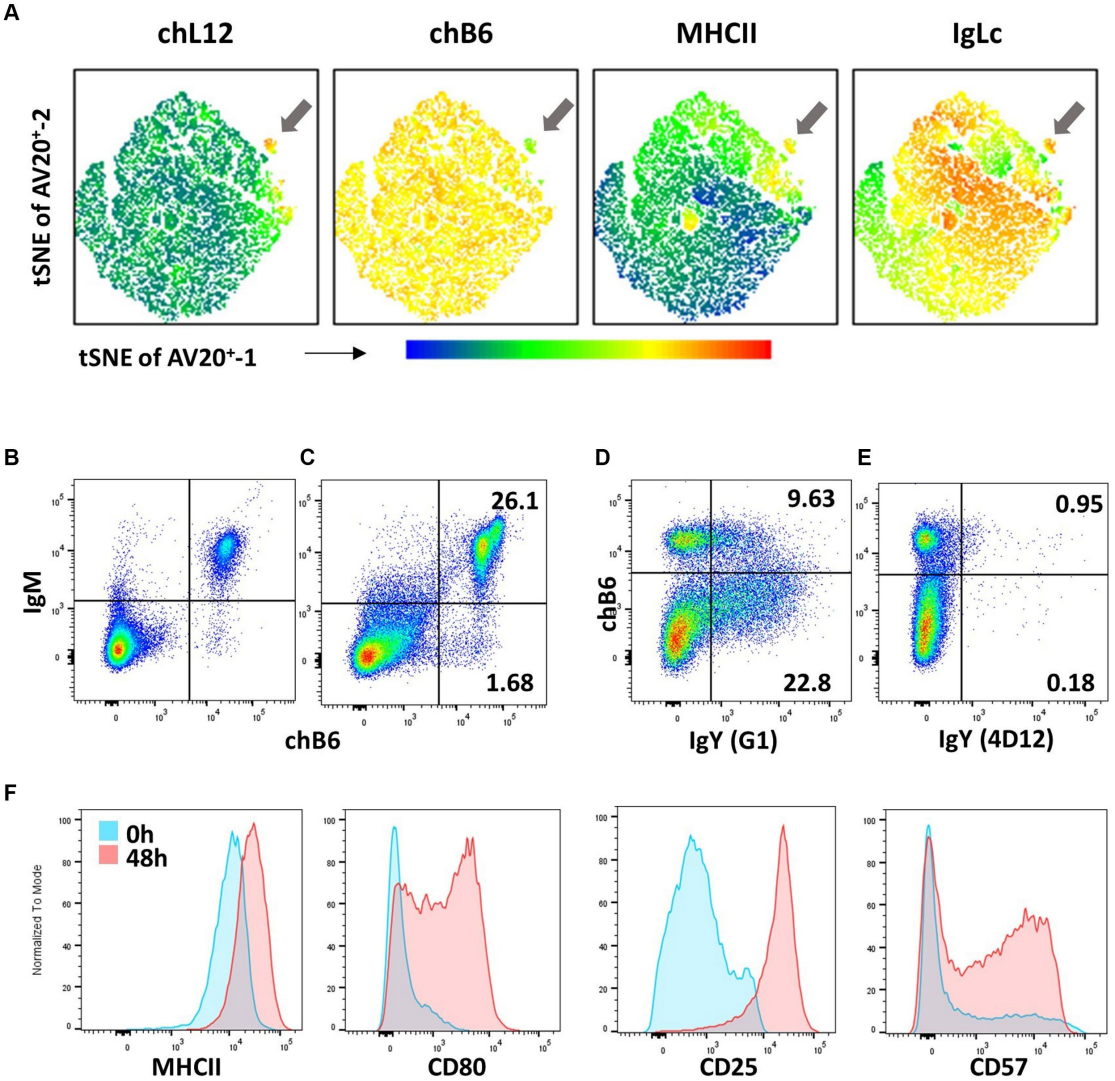
B-cell phenotypes **(A)** Bursa cells from a 5-day-old bird were stained for chL12 (11A9), chB6 (AV20), MHCII (2G11), and IgL (2G1). tSNE analysis with the FlowJo plugin was performed on viable, single, chB6^+^ cells, clearly displaying the phenotype of bursal emigrants (arrow). Leukocytes from the blood **(B)** and spleen **(C)** were stained for chB6 (AV20) and IgM (M1). Plots are gated for viable, single leukocytes. **(D,E)** Recognition of membrane-bound IgY. Spleen cells were stimulated for 6 days with CD40L and IL-10 to induce B-cell proliferation and class switch. Cells were stained with anti-chB6 (AV20) to address undifferentiated B cells and anti-IgY clone G1 **(D)** or anti-IgY clone 4D12 **(E)**. Plots are gated for viable, single leukocytes. **(F)** Immediately after isolation (blue) or after stimulation with CD40L and IL-10 for 48 h (red), splenic leukocytes were analyzed for MHC class II (2G11), CD80 (AV82), CD25 (28–4) and CD57 (HNK-1) expression. Histograms were gated for viable, single chB6+ B cells.

Between ED9 and ED12, a small number of pre-bursal stem cells migrate to the bursa anlage and colonize the lymphoid follicles, where they start to strongly proliferate and diversify their BCR by gene conversion. From ED14 to 18, bursal B cells show a homogeneous expression of chB6, BAFF-R, MHC class II, CD40, CXCR4, and CXCR5 (36, 82, 84, 87, 88) and the initially small percentage of surface IgM-positive cells increases up to 50%. During their differentiation in the bursa, B cells lose CD15s expression and become CD15-positive (85, 86). Around hatch, the first B cells emigrate from the bursa into the periphery. The small fraction (*ca.* 5%) of emigrating cells amongst bursal B cells can be addressed as Ig Light chain^+^, MHC class II^+^, chL12^+^, and CXCR4^Low^ cells (see Figure 5A) (82, 89). ChL12, or the OV antigen, is recognized by mAb 11A9. The nature of the antigen is not known, but it should always be considered that it is an alloantigen, which is not recognised by 11A9 in every chicken line (90).

### 7.3 Peripheral B cells

Post-emigration from the bursa, the immature B cells seed B-cell areas in secondary lymphoid organs, such as the peri-ellipsoidal white pulp in the spleen or the B-cell areas in caecal tonsils. Due to the special structure of the avian spleen, there is no histological discrimination between marginal zone and follicular B cells (91) and, to date, no markers have been described that would assign splenic B-cell subpopulations. All chicken B cells are CD5^+^ (92) hence, CD5-based discrimination of chicken B1 and B2 cells performed in mice and with reservations in humans is not possible. Splenic B cells are quite homogeneously BAFF-R^+^, Ig Light chain^+^, MHC class II^+^, and CD40^+^ (36, 83, 93). Frequently, chB6 expression is not completely homogenous; instead, especially in the spleen, a small immunoglobulin Light chain^+^ fraction expressing higher amounts of chB6 and a higher FSC can be observed (Figures 5B,C) (84). The BCR on the vast majority of cells (>95%) is an IgM isotype, with very few cells expressing a class-switched BCR of IgY or IgA isotype (Figure 5B). Noteworthy, not all commercially available anti-chicken IgY antibodies stain both soluble and membrane-bound IgY. Whilst clone 4D12 works optimally for ELISA and immunohistology, it does not stain membrane-bound IgY. However, anti-chicken IgY clone G1 stains both IgY variants (see Figures 5D,E) (94).

### 7.4 Germinal centre B cells

In the spleen and similarly in all larger secondary and tertiary lymphoid accumulations, encapsulated germinal centres (GCs) can be identified, consisting predominantly of GC B cells. It is important to be aware that in regular spleen preparations (mincing through a strainer), GCs and hence GC B cells will not be present. The GCs strongly stick to the residual artery tree and do not pass through the sieve without further measures. Imamura and colleagues have shown that chB6^+^ GC B cells can be obtained when the splenic artery tree with adjacent GCs is freed of the red pulp and separately digested with collagenase (95). Large GCs with a comparatively thinner capsule, which are not attached to an artery, are found in the caecal tonsils (96) hence, caecal tonsil preparations potentially contain GC B cells. To date, no markers are available to address these cells specifically. However, next to a high expression of chB6, immunohistochemistry of GCs revealed a weak positivity for CD57 (using the cross-reactive anti-human CD57 clone HNK-1) (97). In addition, HNK-1 works in flow cytometry, and as PWP B cells are chB6^+^ CD57^−^, it could serve as a marker to identify GC B cells in cell suspensions. Indeed, despite the lack of dead cells and doublet exclusion, older flow cytometric analysis of caecal tonsil cell suspensions found a chB6^high^ CD57^+^ cell population, which could readily represent GC B cells (97).

### 7.5 Memory B cells

As with GC B cells, due to the lack of markers, it is currently not possible to identify memory B cells by flow cytometry. Here, new techniques like single-cell sequencing of B cells from different tissues and BCR sequencing will certainly help to identify these differentiation stages and potential new markers. Interestingly, scRNA sequencing of chicken blood leukocytes has identified several different B-cell subclusters (18). Though these have so far not been functionally assigned, it highlights the great value of this technique to identify chicken B-cell subpopulations.

### 7.6 Plasma cells

Histologically, plasma cells, the final differentiation stage of B cells, can be identified by their typical morphology with a cartwheel nucleus structure. They have been identified in the spleen and in mucosa-associated lymphoid tissues (HALT, BALT, and GALT). Immunohistochemistry has also shown that in contrast to all other known chicken B-cell stages, plasma cells do not express chB6 or show only very weak staining (47). Downregulation of chB6 has also been shown by flow cytometry when B cells were differentiated toward a plasma cell phenotype *in vitro* by the presence of CD40L (93). Based on single color immunohistochemistry, it is also suggested that splenic plasma cells express CD57, and basic flow cytometry revealed a small population of large cells in the spleen and caecal tonsils expressing chB6^low^ CD57^+^ (97). However, this observation should be verified with further experiments, including additional markers. A rich source for plasma cells may be the Harderian gland, a lacrimal gland in the eye orbit. The gland reacts to intra-ocular vaccination, and it is described that leukocytes, including those with a plasma cell phenotype, can be isolated from the gland (98–101). Immunohistochemistry of the gut reveals a multitude of IgA-positive plasma cells in the lamina propria (LP), and with enzymatic digestion, it is possible to isolate IgA surface-positive cells (98).

Another approach to identify B-cell differentiation stages could be staining for transcription factors. Two key transcription factors for plasma cell differentiation are IRF4 and Blimp1/PRDM1. Whilst IRF4 induces plasma cell differentiation by directing immunoglobulin class switching, proliferation, and survival, BLIMP1 acts as a transcriptional repressor that represses B-cell features (102). For the rat anti-human-IRF4, clone 3E4 cross-reaction with porcine cells has been demonstrated (103) and preliminary data suggest cross-reaction with chicken cells (personal communication, Dr. W. Gerner). At least for one anti-Blimp1 antibody (mAb rabbit anti-human Blimp1, clone C14A4), cross-reactivity with the chicken protein in Western blots was demonstrated (104), and in mammals, this antibody was used in flow cytometry.

### 7.7 Post-bursal stem cells

In contrast to mammals, where B-cell production in the bone marrow can be a lifelong process, the bursa Fabricii, and hence the chicken’s primary B-cell organ, involutes with sexual maturity (78). As analysis of older birds and studies with bursectomised birds have clearly shown the establishment of a bursa-independent dividing B-cell pool post-hatch, it is postulated that after bursa involution, the peripheral B-cell pool is maintained by post-bursal stem cells (89). Whilst after bursal emigration all B cells are chL12^+^, with increasing age, a chB6^+^/chL12^−^ B-cell population becomes detectable in the spleen, which might represent these cells (105). As chL12 detects an alloantigen, it may not be useful for birds lacking the allele. With the availability of new techniques and markers, these cells can now be further analyzed.

### 7.8 Activation markers

As antigen-presenting cells, all B cells are MHC class II^+^, but CD40L stimulation and mimicking T cells help further increase MHC class II (93). Whilst freshly isolated B cells from bursa, PBL, and spleen are CD80^−^, *in vitro* activation of B cells leads to strong upregulation of CD80 and also strongly increases CD25 expression (106, 107). As mentioned, regular spleen cell preparations do not contain GC B cells, so it is possible that, like human B cells, CD80 is a marker for activated and dividing chicken GC B cells. Up to one-third of freshly isolated splenic B cells are already CD57^+^ (97). This fraction is doubled by *in vitro* stimulation. Overall, activation of B cells can lead to an increase in already existing marker expression on all cells (MHC class II, CD25), complete *de novo* expression on a subset of B cells (CD80), or expression on an increased fraction of cells (CD57) (Figure 5F).

## 8 T cells

T-cell progenitors in the bone marrow express several markers like c-kit, HEMCAM, BEN, αIIbβ3, ChT1, MHC class II, and CD44 (108), and they colonize the thymus in three waves (first from paraaortic foci starting at ED6, second at ED12, and third at ED18, the two latter from bone marrow) (109). Embryonic thymocytes expressing the TCR γδ can first be detected around ED12, whereas cells expressing TCRα/vβ1 are not present until ED15 and TCRα/vβ2 around ED18 (110, 111). As in mammals, avian extra-thymic T lymphocytes all express the T-cell marker CD3. The common commercial antibody clone CT-3 recognises an extracellular domain of the chicken CD3 molecule (112). However, CT-3 staining may or may not give optimal separation between negative and positive populations, especially in whole blood. Moreover, even in isolated PBMCs, the staining can be influenced by, e.g., the chicken breed or cell activation status (7). In addition to CT-3, commercial anti-human CD3ε (clone CD3-12) antibodies exist where cross-reaction with chicken CD3 has been shown for intracellular staining (113). Reports exist on CD3 polymorphism, and another anti-chicken CD3 antibody, clone AV36, supposedly recognises a variable epitope and did not bind to splenocytes from the NARF C.B12 (B12 haplotype) inbred chicken line (114), whereas clone CT-3 could detect C.B12 splenocytes. In addition to CD3, avian T-cell subsets can be defined according to the expression of T-cell receptor variants. Avian homologs of the mammalian γδ and αβ TCR exist, but two variants of the latter were shown to differ in the variable regions in the β chain [encoded by either Vβ1 or Vβ2 genes (115)].

### 8.1 αβ T cells

Avian T-cell subsets expressing the variants of the αβ TCR can be identified by staining with the commercial clones TCR2 (TCR αVβ1) and TCR3 (TCR αVβ2), respectively (116). TCR2^+^ and TCR3^+^ subsets differ in ontogeny and tissue distribution, with TCR2^+^ cells in general being more abundant than TCR3^+^ cells (117). Functional differences between TCR2^+^ and TCR3^+^ subsets are poorly described, but interestingly, TCR2^+^ cells but not TCR3^+^ cells migrate to the chicken intestine, hence being of importance to mucosal IgA production (118, 119). Several subsets of αβ T cells can be identified by staining for the co-receptors CD4 and CD8 (120). As opposed to chicken CD4 and CD8β, the chicken CD8α gene is polymorphic (121). Several antibodies exist (CT-8, 3–298, EP72, AV12-14) recognising chicken CD8α, but the 3–298 clone may be superior in being the only commercial reagent recognising the majority, if not all, of CD8α variants (122). In contrast to CD8α, only a single commonly used CD8β antibody exists (EP42). Within the αβ T-cell population in, e.g., peripheral blood, the following subsets exist: CD4^+^ CD8αα^+^, CD4^+^ CD8αβ^+^, and CD4^−^ CD8αβ^+^, in addition to the less well-characterized subsets of CD4^−^ CD8αα^+^ (123) and CD4^+^CD8αβ^+^ cells (124).

### 8.2 Cytotoxic T cells

Chicken cytotoxic T lymphocytes (CTL) recognize peptides presented by MHC I molecules and show cytotoxic activity (125, 126). Interestingly, CD3^+^CD8^+^ cells in peripheral blood usually express the CD8αβ isoform, but a CD8αα-positive subset also exists and may expand, e.g., during viral infection (127). A common CTL assay in mammals is based on the detection of transient expression of lysosomal-associated membrane glycoproteins (LAMPs) on the cell surface. Cytotoxic activity via the perforin–granzyme pathway occurs in pre-formed lytic granules surrounded by lipid bilayers containing LAMPs that are fused with the plasma membrane. Hence, the LAMP-1 (CD107a) degranulation assay described above can also be used in studies of chicken cytotoxic T cells. Within the αβ T-cell population (αVβ1+ splenocytes), both CD8αα^+^ and CD8αβ^+^ showed potential to degranulate *in vitro* upon phorbol myristate acetate (114).

In addition to the granzyme/perforin killing pathway, evidence exists of a Fas/FasL pathway in chickens (128) and an anti-human FasL antibody (CD178, clone SB93a) was shown to cross-react with the chicken FasL by immunohistochemistry (129) but its suitability for flow cytometry is to be determined. A common parameter reported in relation to CTL responses is the production of IFN-γ. A number of monoclonal antibodies directed against chicken IFN-γ exist. Some only recognize the recombinant protein they were raised against, and others perform well in ELISA but are not suited for flow cytometry. Induction of IFN-γ production in splenocytes or PBMC by mitogens or specific antigens is reported to be detected by using antibody clones: 2B7, 11G5, 7E3, 12F12 (130, 131), mAb80 (132), and a rabbit polyclonal anti-chIFN-γ reagent (133). Clones 12F7, 12D4 (130) and EH9 (134) seem less suitable for intracellular staining (ICS) and flow cytometry. The anti-chicken IFN-γ antibody (clone 5C.123.02/08) from the commercial chicken IFN-γ Invitrogen ELISA kit works for intracellular staining (ICS) of the recombinant protein expressed by CHO cells (133). However, although some report staining of the native protein using these antibodies, others observe only weak staining with the ELISA reagents (135) or fail to reproduce even a dim signal (136). Additional clones MT6C2 and MT7C1 from Mabtech, as well as four Chinese clones, were found not suitable for ICS (136, 137). Unfortunately, the two superior antibody clones for intracellular staining and flow cytometry, mAb80 and 5G11, are not commercially available.

### 8.3 T helper cells

**Figure 6:**
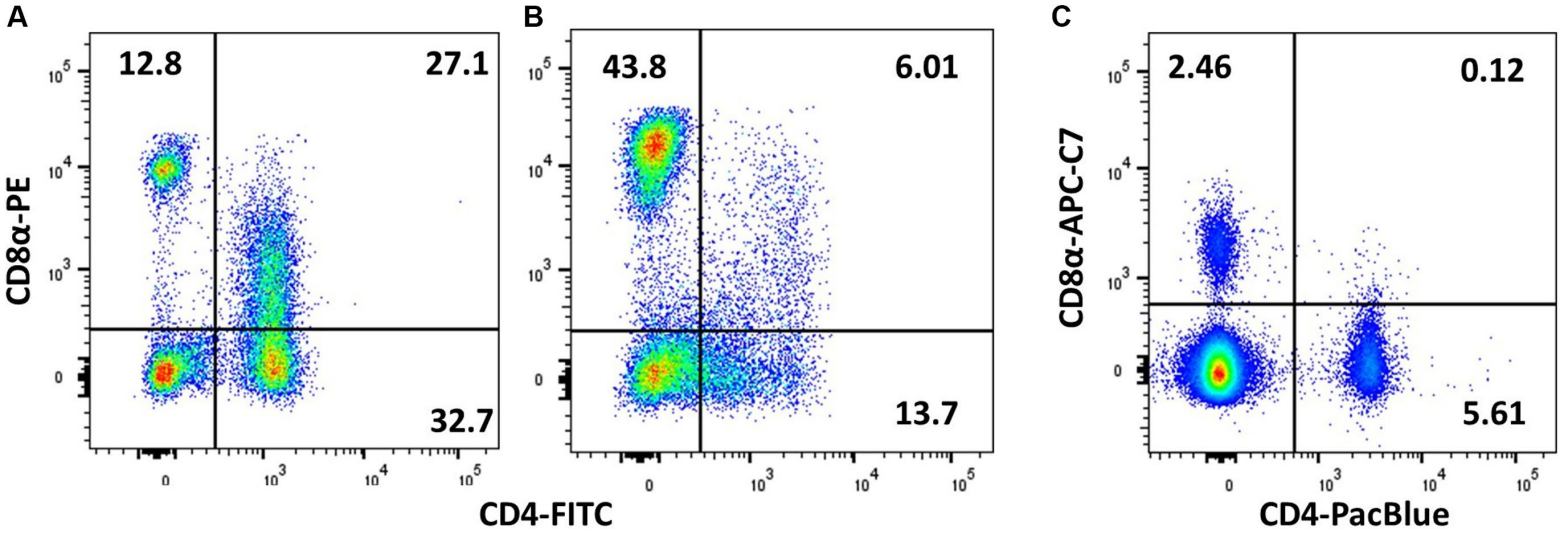
Demonstration of CD4/CD8 double-positive cells is affected by the choice of fluorochrome and the individual animal. PMBCs from adult birds were stained with TCRγδ (TCR1), CD4 (CT-4), and CD8α (3–298), and plots are shown for viable singlets and TCR1^−^ lymphocytes. **(A)** Chicken with high level of peripheral CD4 + CD8+ cells and **(B)** a low level of double-positive cells. **(C)** Chicken with high level of CD4 + CD8+ cells, which is not picked up by the less bright APC-Cy7 staining of CD8α.

Several reagents recognising chicken CD4 are available, e.g., the clones CT4, EP96, AV29, and 2–35. In other species, CD4 may also be expressed by monocytes, but this is not the case in chickens (138). However, a small CD3^−^ CD4^+^ NK population (with slightly higher FSC/SSC than resting lymphocytes) is sometimes identified in peripheral blood (54) and hence at least TCRαβ or CD3 in combination with CD4 should be used to identify chicken Th cells. Interestingly, some lines of chickens have a high abundance of CD4^+^CD8^+^ double-positive TCRαβ^+^ lymphocytes, and there is a genetic influence on levels in peripheral blood but not necessarily in the intestine (121, 123, 138). The double-positive subset exists either as CD4^+^CD8αα^+^ or CD4^+^CD8αβ^+^ with a dimmer CD8 signal than CD4^−^ CD8^+^ cells (127); hence, using bright fluorochromes for CD8 detection is crucial to obtain good separation between CD8^−^, CD8^dim^, and CD8^Hi^ subpopulations (Figure 6).

In mammals, major Th subsets can be differentiated by intracellular staining for the transcription factors T-bet (Th1), GATA-3 (Th2), RORγt (Th17), and Foxp3 (Treg). To the best of our knowledge, no reagents are available for staining important chickens Th transcription factors, despite the obvious value of developing such reagents for flow cytometry. The success rate of identifying cross-reacting mammalian reagents is generally poor for chicken surface markers (139, 140), but for highly conserved intracellular proteins such as transcription factors, the chances may be higher. However, testing of two widely used anti-murine Foxp3 clones, FJK-16 s and MF-14, proved they were unsuitable for Foxp3 detection in chicken cells (141).

The basic Th1 response system appears to be conserved in chickens (142), and CD4^+^ cells producing IFN-γ are often interpreted as Th1 cells. Indeed, the Th1/Th2 paradigm was early on made probable through gene expression analysis of tissue from Newcastle disease virus and *Ascaridia galli*, infected chickens, respectively (143). However, flow cytometry studies addressing production of multiple cytokines and linking Th1 or Th2 profiles to, e.g., TCR αVβ1 or TCR αVβ2 expression are still missing. The limited quality of the chicken cytokine antibodies is often a problem that has inspired the use of alternative methods such as identification of intracellular cytokine RNA by the PrimeFlow^™^ system (130).

Putative chicken regulatory T cells that can suppress T-cell proliferation *in vitro* were identified by co-expression of CD4 and CD25 (144). However, the CD4^+^ CD25^+^ population includes other subsets than just Tregs. More recently, *FOXP3* was identified in the chicken genome, and Foxp3 mRNA was shown to be abundant in CD4^+^ CD25^+^ in contrast to CD4^+^ CD25^−^ subsets in the spleen and caecal tonsils (141). The gene expression studies identified two CD4^+^ CD25^+^ subsets where the cells expressing high levels of IL-10 and Foxp3 were suggested as mature Tregs, whereas cells expressing low levels of IL-10 in combination with IL-2 were rather activated Th cells (141). Staining with CD4 and CD25 will hence provide a mixed population, and the production of chicken Foxp3 antibodies is expected to give better opportunities for studying Treg subsets in the future. Several chicken CD25 antibodies exist where AV142 and the bivalent human recombinant Fab AbD13504 are widely used. In addition, clones like 6C9, chCD25-32, and chCD25-54 exist (145), as well as 28–4, which was originally described as an NK cell marker but later identified as recognising CD25 (46).

Various *in vitro* and *in vivo* models have shown chicken IL-17 mRNA gene expression and suggested the presence of Th17 cells (146–149) but only recently have monoclonal antibodies useful for ICS been developed, namely the 1E7 clone recognising IL-17F (with slight cross-reactivity to IL-17A) and the two IL-17A-specific clones 9F11 and 10D5 (150). The IL-17 antibodies were all able to stain a small population of CD4^+^ splenocytes upon PMA activation. The 10D5 clone was furthermore used to show that IL-17A was primarily expressed by CD3^+^ CD4^+^ T cells in the spleen and PBMC, but staining of smaller subsets of γδ T cells was also evident (151). Interestingly, IEL staining patterns were slightly different with IL-17A^+^ cells largely CD4^−^, CD8^−^, and TCR1^−^ but for the most part expressing CD3 and CD25 (150). In addition, the same antibody was used to show weak signals of IL-17A in lung T-cell populations (131).

### 8.4 γδ T cells

The commercially available antibody TCR1 recognises the TCRγδ variant, and hence all TCR1^+^ cells are actually γδ T cells (111). However, whether all γδ TCRs indeed express the TCR1 epitope is still not proven but might be solved in the near future with the availability of new TCR sequencing protocols (152). As shown by frequency within a lymphocyte gate, γδ T cells are abundant in peripheral blood, immune organs, and bone marrow, and a CD8^+^ subset is often seen in the lung and spleen (137), as well as in the intestinal mucosa (153). In the intestine, γδ T cells are present both in the intraepithelial and the lamina propria areas, and interestingly, in addition to the CD4^−^ CD8^−^, CD4^−^ CD8αα^+^, and CD4^−^ CD8αβ^+^ populations, the presence of an additional small CD4^+^ CD8^−^ population has been suggested. The CD8^+^ population has received much attention and has been reported as being slightly larger in size and more prone to activation by mitogens (154). Interestingly, chB6-positive intraepithelial lymphocytes in the small intestine contain a population of TCR1^+^ cells, as shown in Figure 3.

Some molecules are differentially expressed between γδ T cells and αβ T cells but cannot be used as unique lineage identifiers. For example, CD5 was shown to be expressed on virtually all CD4^+^ αβ T cells and on the majority of γδ T cells, but the mean fluorescence intensities were low/intermediate on the γδ T cells isolated from spleen and peripheral blood (92). The CD5 antibody clone 2–191 unfortunately appears to be discontinued. CD28 is another molecule expressed on virtually all CD4^+^ αβ1 T cells but is absent from the majority of γδ T cells; however, by using the clone MoAb 2–4, a small subset was found to be CD28^+^ (mostly CD8α^+^ but also a minor CD8^−^ population) (155). The clone used by Koskela et al. may no longer be accessed, but clone AV7 recognises CD28 and is commercially available (156).

Data from scRNA-seq suggest the presence of multiple γδ T-cell subsets that may represent either phenotypic subsets or differentiation and activation states (18). A range of molecules may be differentially expressed by various γδ T subsets, but a comprehensive multiparameter immunophenotyping study has not yet been published. As the percentage of TCR1+, TCR2+, and TCR3^+^ cells does not completely add up to 100% of the CD3 population, there may be a small yet unidentified T-cell subpopulation. Hence, interesting surface marker antibodies for a future multiparameter staining panel may include TIM4 (ROS-JH9 (60)), SLAMF4 (8C7 (35)), CD25 (AbD13504 (157)). Moreover, chicken γδ T cells have the ability to secrete a number of cytokines such as IFN-γ, IL-17A, IL-6, IL-10, and IL-13 (151, 158, 159), and including cytokine staining in multiparameter staining is of value to characterize various γδ T-cell subsets.

### 8.5 T-cell activation and memory cell markers

Extensive knowledge about T-cell activation and memory cell markers is still lacking in the avian research field. In mammals, activated proliferating T cells express several molecules that are expressed to a lesser extent or even absent on resting cells, including various chemokine receptors, adhesion molecules, co-stimulatory molecules, and MHC antigens (160). The same appears to be the case for chicken lymphocytes, but most of the published data include observations made using *in vitro* polyclonal/mitogen-stimulated cells rather than *in vivo* activated cells from infected animals. For example, *in vitro* ConA-induced T-cell proliferation of PBMC confirmed CD25 and MHC class II as T-cell activation markers for both CD4^+^ and CD8α^+^ cells and CD28 only for CD8α^+^ cells when looking at activation marker-positive frequencies of cells (6). Interestingly, most of the tested putative activation markers (e.g., CD44, CD45, CD25, and CD28) showed increased surface expression (mean fluorescent intensity, MFI) over time, whereas the MFI of MHC class II was upregulated only 24-h post-stimulation, followed by MFI downregulation, especially in CD8α^+^ T cells, where the MFI stayed below baseline from 48- to 96-h post-stimulation.

In mammals, constitutive expression of MHC class II is confined to professional antigen-presenting cells, including DCs, B cells, monocytes, and macrophages, and upon activation, MHC class II expression (all isotypes) is also seen on the surface of T cells in various species except in mice (161). The Naghizadeh chicken PBMC study mentioned above (160) showed virtually no MHC class II expression pre-stimulation, but 24-h post-stimulation, it was readily induced in 20–25% of the CD4^+^ and CD8α^+^ cells. Although the CD8α^+^ antibody would have picked up a small subset of γδ T cells, the majority of γδ T cells (CD8^−^) were excluded from the mentioned study. Interestingly, others have reported that the majority of γδ T cells in peripheral blood express MHC class II even in a resting state, and increased MFI was shown in an *in vivo* experiment where chickens were provided high doses of Ulvan in their drinking water (162). CD25 is constitutively expressed on a subset of γδ T cells but is also described as an activation marker because frequencies of CD25^+^ cells within the γδ T population are increased upon activation with, e.g., *Salmonella* and *Eimeria* (124, 157). Furthermore, chickens immunized with the model antigen mycobacterial purified protein derivative or *Mycobacterium tuberculosis* sonicate showed increased frequencies of CD28^+^ γδ T-cell frequencies combined with increased surface expression of CD28, CD5, CD25, and MHC class II (155).

CD57 (clone HNK-1) has been identified as a B-cell activation marker (44, 97, 163, 164). We have shown higher degranulation of CD57^+^ CTLs than of CD57^−^ CTLs, which supports CD57 as chicken T-cell activation marker (165). Furthermore, frequencies of CD57^+^ cells increase within both αβ and γδ T-cell populations in PBMC stimulated with mitogens or anti-CD3 (T. Dalgaard, manuscript in preparation). Several additional potential activation markers are available, but their expression on activated chicken T cells is poorly characterized. Examples include CD276 (AV95/EH7), CD30 (AV37 (166)), CRTAM (8A10 (167)), and GITR (9C5 (168)). In addition to activated T cells, chicken memory cells are also poorly defined in terms of phenotypes. Despite the fact that different CD45 isoforms exist (29), the equivalents of CD45RA and CD45RO have not been identified in chickens. CD127 is the α chain of the IL-7 receptor, and in mammals, it is differentially expressed depending on T-cell differentiation state (naïve, effector, memory) (169). A monoclonal antibody against chicken CD127 exists (clone 8F10E11) and was used to show that the majority of CD4^+^ cells in the peripheral blood and spleen of healthy animals expressed CD127. In contrast, only 10–60% of the CD8α^+^ cells expressed CD127, and the frequency declines with age (170). To establish the value of this marker for the discrimination of T-cell differentiation stages, CD127 staining must be further investigated, e.g., on tetramer-positive cells and in conjunction with other activation or memory cell markers. In other species, CD44 expression is higher on effector and memory T cells as compared to naive. This is not yet established for chicken cells, although some studies suggest increased CD44 expression in the memory stages of *in vivo* vaccine or challenge experiments (5, 171).

### 8.6 Antigen-specific T cells

To evaluate host–pathogen interaction and vaccine responses, it is important to understand the role of antigen-specific T cells. Few avian MHC multimer flow cytometry reports exist (172, 173) and the reagents are not commercially available in various MHC–peptide combinations as they are for human and mouse models. Hence, most analyses of avian T-cell biology have relied on proliferation, degranulation, or cytokine detection in PBMC or splenocytes after antigen re-stimulation *in vitro*/*ex vivo* for quantitative and qualitative studies of antigen-specific T-cell responses. Interestingly, a subset of activation markers in mammals are exclusively expressed by T cells activated via MHC–TCR interaction, like the commonly used marker CD154 (CD40L) for antigen-activated T cells in humans and mice (174, 175). Transient CD154 surface expression or intracellular expression stabilized with the secretion inhibitor Brefeldin can be exploited for enrichment of antigen-specific T cells before further analysis (176, 177). An avian orthologue of mammalian CD154 exists, and the gene was identified and a set of monoclonal antibodies named AV71-76 were generated (UK Immunological Toolbox). Initial analysis of splenocytes activated by PMA/IO showed binding of the antibodies and suggested CD154 expression on activated T cells. In this early study, it was evident that the IgG1 antibodies AV71 and AV74 bound weakly to CD154, whereas the IgG2a antibodies AV72, AV73, and AV75 showed slightly higher affinity; however, further in-depth analysis is warranted (36).

## 9 Pitfalls

In the current study, we have focused on the spleen and peripheral blood; hence, this is not a comprehensive overview of cell subsets found in other tissues and organs. Moreover, it is important to note that both absolute numbers and relative frequencies of cellular subsets are influenced by, e.g., chicken breed and age (7). In addition, even the same tissue can give different results with different isolation techniques (choice of enzyme, slow speed/Ficoll gradient, etc.). Careful optimisation is therefore needed for each application, with a special focus on understanding potential epitope degradation by the digest or fixation protocol in question. Moreover, fixed samples may require different gating as compared to fresh samples, as fixation may compress FSC/SSC profiles and create problems with autofluorescence, which is, e.g., very pronounced in PFA-fixed thrombocytes. Biotinylated antibodies are often used in flow cytometric experiments with chickens due to the limited choice of directly conjugated antibodies. Since avidin is a biotin-binding protein with a possible antimicrobial effect and is upregulated after stress and infection (178), appropriate controls must be included. To check for non-specific biotin binding, an irrelevant biotinylated antibody (a non-chicken target antigen) should be included. Another well-known technique that works well for mammalian samples is lysis of erythrocytes before flow cytometry acquisition. Several groups have published data where attempts to lyse erythrocytes were made, e.g., by prolonging exposure to commercial lysis buffers with suboptimal results. However, whereas this strategy may not be possible for peripheral blood, it may work for tissues such as the bone marrow, where erythrocyte content is lower. In any case, careful validation and assessment of any effect on the phenotype and function of the remaining leukocytes is necessary.

In-depth transcriptome analysis will certainly identify further subpopulations and potential markers, and flow cytometry and cell morphology will not necessarily match with them. Hence, carefully controlled cross-validation of all sources should be a prerequisite for a successful combination of all techniques.

## Author contributions

SH: Conceptualization, Visualization, Writing – original draft, Investigation, Funding acquisition, Methodology, Writing – review & editing. KS: Conceptualization, Writing – original draft, Visualization. LV: Conceptualization, Visualization, Writing – original draft, Funding acquisition, Writing – review & editing. TD: Conceptualization, Visualization, Writing – original draft, Investigation, Methodology, Writing – review & editing.
